# Deep learning approach to the estimation of the ratio of reproductive modes in a partially clonal population

**DOI:** 10.18699/vjgb-25-50

**Published:** 2025-06

**Authors:** T.A. Nikolaeva, A.A. Poroshina, D.Yu. Sherbakov

**Affiliations:** Limnological Institute of the Siberian Branch of the Russian Academy of Sciences, Irkutsk, Russia Novosibirsk State University, Novosibirsk, Russia; Limnological Institute of the Siberian Branch of the Russian Academy of Sciences, Irkutsk, Russia; Limnological Institute of the Siberian Branch of the Russian Academy of Sciences, Irkutsk, Russia Novosibirsk State University, Novosibirsk, Russia

**Keywords:** deep learning, convolutional neural network (CNN), Hardy–Weinberg equilibrium, partially clonal population, microsatellites, глубокое обучение, сверточная нейронная сеть (CNN), равновесие Харди–Вайнберга, частично клональная популяция, микросателлиты

## Abstract

Genetic diversity among biological entities, including populations, species, and communities, serves as a fundamental source of information for understanding their structure and functioning. However, many ecological and evolutionary problems arise from limited and complex datasets, complicating traditional analytical approaches. In this context, our study applies a deep learning-based approach to address a crucial question in evolutionary biology: the balance between sexual and asexual reproduction. Sexual reproduction often disrupts advantageous gene combinations favored by selection, whereas asexual reproduction allows faster proliferation without the need for males, effectively maintaining beneficial genotypes. This research focuses on exploring the coexistence patterns of sexual and asexual reproduction within a single species. We developed a convolutional neural network model specifically designed to analyze the dynamics of populations exhibiting mixed reproductive strategies within changing environments. The model developed here allows one to estimate the ratio of population members who originate from sexual reproduction to the clonal organisms produced by parthenogenetic females. This model assumes the reproductive ratio remains constant over time in populations with dual reproductive strategies and stable population sizes. The approach proposed is suitable for neutral multiallelic marker traits such as microsatellite repeats. Our results demonstrate that the model estimates the ratio of reproductive modes with an accuracy as high as 0.99, effectively handling the complexities posed by small sample sizes. When the training dataset’s dimensionality aligns with the actual data, the model converges to the minimum error much faster, highlighting the significance of dataset design in predictive performance. This work contributes to the understanding of reproductive strategy dynamics in evolutionary biology, showcasing the potential of deep learning to enhance genetic data analysis. Our findings pave the way for future research examining the nuances of genetic diversity and reproductive modes in fluctuating ecological contexts, emphasizing the importance of advanced computational methods in evolutionary studies.

## Abstract (ru)

Генетическое разнообразие биологических объектов, таких как популяции, виды и сообщества, является важнейшим источником информации для понимания их структуры и функционирования. Однако многие экологические и эволюционные проблемы возникают из-за того, что наборы данных содержат относительно небольшое количество выборок, что затрудняет использование традиционных методов анализа. В связи c этим наше исследование предлагает новый подход, основанный на глубоком обучении, для решения одной из самых актуальных задач эволюционной биологии – поиска баланса между половым и бесполым размножением. Половое размножение часто приводит к нарушению выгодных комбинаций генов, которые были отобраны в процессе эволюции. С другой стороны, бесполое размножение позволяет организмам быстрее размножаться без участия самцов, эффективно поддерживая полезные генотипы. Исследование посвящено изучению закономерностей сосуществования полового и бесполого размножения в рамках одного вида. Мы разработали специальную сверточную модель нейронной сети, предназначенную для анализа динамики популяций, которые демонстрируют смешанные репродуктивные стратегии в изменяющихся условиях. Эта модель позволяет оценить долю потомков репродуктивного размножения, если эта доля остается постоянной в течение достаточного периода времени, в популяциях, состоящих из постоянного числа организмов, с использованием мультиаллельных признаков, таких как микросателлитные повторы. Результаты показали, что модель с точностью 0.99 оценивает соотношение репродуктивных режимов, эффективно справляясь с трудностями, связанными с небольшими выборками. Более того, когда размерность обучающего набора данных соответствует фактическим данным, модель быстрее достигает минимальной ошибки, что подчеркивает важность подбора структуры набора данных для точности предсказаний. Эта работа вносит значительный вклад в понимание динамики репродуктивной стратегии в эволюционной биологии, демонстрируя потенциал глубокого обучения для улучшения анализа генетических данных. Наши результаты открывают двери для будущих исследований, посвященных тонкостям генетического разнообразия и способам размножения в изменчивых экологических условиях, подчеркивая важность современных вычислительных методов в эволюционных исследованиях

## Introduction

Genetic diversity of biological entities such as populations, species, species communities is the main source of information allowing one to make numerous conclusions about their setup and functioning (Korfmann et al., 2023). Hence, the variety of sampling methods and ways of subsequent experimental data processing have been developed. In contrast to big data applications, where sample sizes typically exceed minimal requirements for robust conclusions, certain problems rely on limited and hard-to-acquire datasets, which complicates processing.

Deep learning has been applied successfully in population genetics in order to study various microevolutionary processes. A recurrent neural network model has been developed to predict recombination maps (Adrion et al., 2020), identify possible cases of positive natural selection (Anders, Korn, 1999; Eğrioğlu et al., 2008) and to estimate the time since the nearest common ancestor (Montinaro et al., 2021). A good predictive effectiveness on simulated data has been shown (Korfmann et al., 2023).

Neural networks were used to elucidate the demographic history of an individual population using genomic data without any preliminary knowledge of the recombination rate (Sanchez et al., 2021). In this study, the authors showed that network architecture is crucial for its performance. A poor design could lead to overfitting and loss of information.

When SNP frequencies were analyzed using MLP (multilayer perception), it led to high prediction errors, since the genomic information was encoded as a simple set of values where the order did not matter, and thus the information provided by the data structure was not used. The MLP configuration has several disadvantages for SNP analysis: (a) the number of estimated network parameters is large, which can lead to an increase in model training time; (b) MLP can extract data geometry only by training, without a guarantee that it will study the spatial structure of the genome. But MLP still works much better than random assumptions or constant prediction (by 32 %) (Sanchez et al., 2021).

In this paper, we apply a deep learning-based approach to one of the most intriguing questions of evolutionary biology: the balance between sexual and asexual reproduction (Schön et al., 2009; Baer, 2020; Otto, 2021; Cohen, Marron, 2023). Sexual reproduction can destroy favorable combinations of genes supported by selection, while the asexual one allows to reproduce twice as fast, since there is no need to produce males for continuous reproduction, and preserve favorable genotypes (Barton, Charlesworth, 1998; Gutiérrez-Valencia et al., 2021).

There are various patterns of coexistence of sexual and asexual reproductive modes in a single species. The sexual and asexual organisms belonging to the same species coexist in the same population, either alternating throughout their life cycle or in spatially or temporarily isolated subpopulations (Tagg et al., 2005; Rossi et al., 2007). Exclusively asexual vertebrates are usually closely related to sexually reproducing species (Janko et al., 2007; Schurko et al., 2009).

Asexual lines (clones) can develop by various mechanisms (spontaneous, contagious or infectious origin, hybridization) from ancestral sexual species (Avise et al., 1992), but the mechanisms of transition may be extremely diverse (Thielsch et al., 2012; Poroshina, Sherbakov, 2023). In order to analyze the exact population processes in organisms able to follow both ways of reproduction, one must be able to estimate the population-wide ratio of reproductive modes. Computer modeling previously allowed us to show that it is possible to do using distortions from equilibrium frequencies of microsatellite alleles (Messer, 2016). Here, we describe the development and testing of a deep learning model designed to study the dynamics of populations with a mixed type of reproduction in a changing environment

## Methods

Experimental data. The experimental data were taken from a published article and represent sets of allele lengths of microsatellites from 44 natural populations of Daphnia cucullata, D. galeata and D. longispina (1715 individuals) expressed in nucleotide pairs (Thielsch et al., 2012). The lengths of microsatellites are converted into matrices reflecting the frequency of occurrence of alleles and analyzed in this form by a neural network.

Simulated data. The training data were generated by a modified version of the Wright–Fisher model (WF), considering a mixed breeding strategy in a population (Messer, 2016). The model describes a population with discrete, nonoverlapping generations. In each generation, the entire population is replaced by the offspring of the previous generation. The parents are selected by random sampling with substitution. In a haploid population of constant size N, the probability that an allele present in i individuals will be present in j individuals in the next generation follows the binomial probability:

**Formula. 1.**
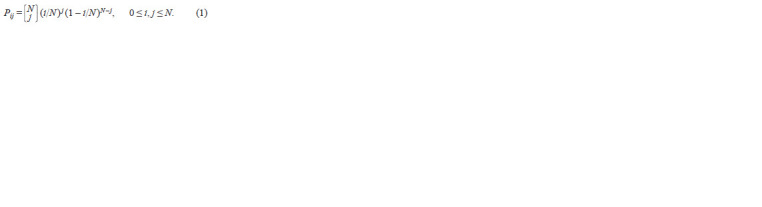
Formula1

The transition probabilities Pij determine the Markov process with discrete time in the space of allele frequencies

**Formula. 2.**
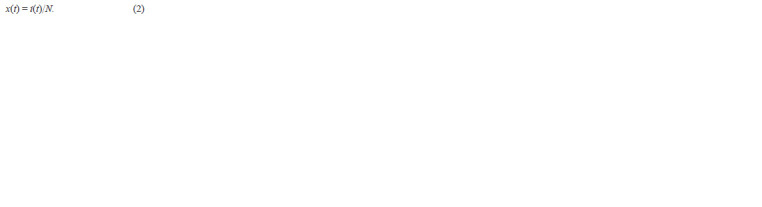
Formula2

The expected frequencies of alleles remain constant across generations, whereas the variance for each generation is:

**Formula. 3.**
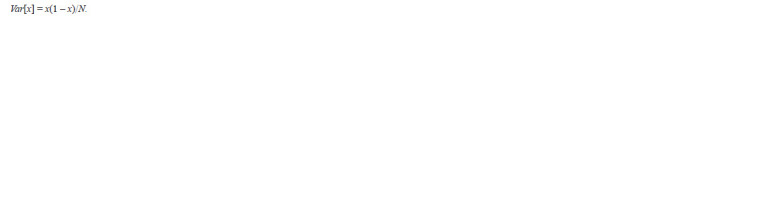
Formula3

The probability that an allele will eventually become fixed is simply its initial frequency. In particular, the probability of fixing a new mutation present in a single copy is 1/N (Ratner, 1972).

Models of genotype distributions resulting in different reproductive modes. If all allele and gene combinations are believed to be of the same adaptive value and the conditions for the WF model are fulfilled, in a sufficiently big population reproducing exclusively sexually the Hardy–Weinberg equilibrium has to be true. In its traditional form, it describes a single locus having two alleles. For this study, we need an expanded model describing equilibrium for multiallelic loci which would be suitable for multiallelic microsatellites markers. Thus, for a gene having m alleles (for microsatellite markers m > 2), an array of allele frequencies P = [ p1, …, pM] and ΣM pi = 1 i = 1 , where M is the number of alleles. The equilibriumprobabilities of diploid genotypes will be

**Formula. 4.**
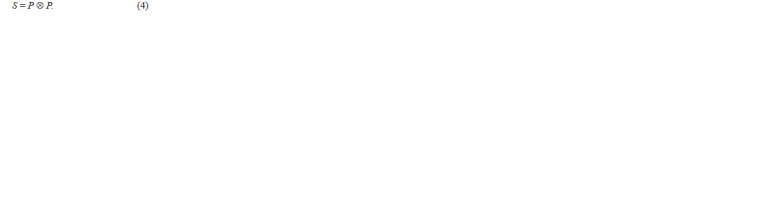
Formula4

In matrix shape:

**Formula. 5.**
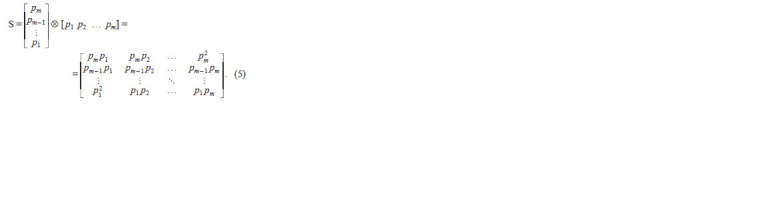
Formula5

And Hardy–Weinberg equilibrium will be:

**Formula. 6.**
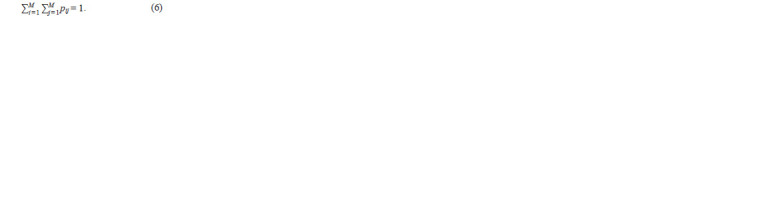
Formula6

And, according to the WF model, it will hold for generations. In case of asexual reproduction, all ancestors of a given organism will inherit its genotype unless a mutation will transform the ancestral allele into a different one. It is important to note that we assume a fixed number of allowed alleles M, possibly different for each polymorphic locus; therefore, no mutation may increase M and frequencies of alleles will be:

**Formula. 7.**
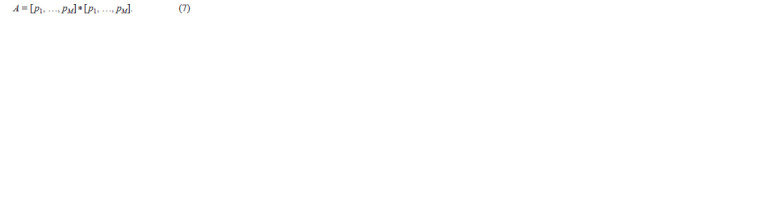
Formula7

Assuming that the ratio of organisms resulting from asexual reproduction to the ones resulting from sexual reproduction is α, the genetic setup of a population with two coexisting reproduction strategies will be:

**Formula. 8.**
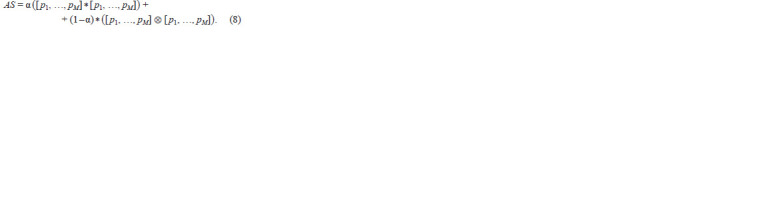
Formula8

Neural network architecture and training. Two sources of noise in real world data have been modeled. Sampling error was mimicked by substituting probabilities of genotypes with their frequencies sampled from a small set of organisms. These frequencies were then converted to probabilities and used for training the network. The resulting values deviate from the expected pattern because of the small sample size.

Possible reasons for additional noise may include misidentification of samples, pipetting mistakes etc. They were simulated by the addition of a random value sampled from a normal distribution with average set to 0 and standard deviation set to 0.05 or any other sufficiently small value.

Neural networks are trained using a matrix of dimension m × n, where m is the number of different alleles of a gene, n is the number of genes, and the element of the matrix aij is the frequency of occurrence of a combination of the i-th and j-th alleles

The training set was obtained by repeating simulation of genotype distributions at different α for n genes, for different numbers of alleles Mi for each gene. The allele frequencies were sampled from a uniform distribution and then the genotype frequencies were obtained using (5).

**Fig. 1.**
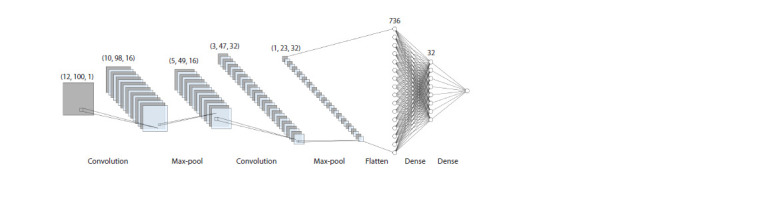
The structure of the neural network. A convolutional neural network contains two external and six internal layers: two convolution layers followed by a max-pooling one, a flattening layer and two dense layers.

A convolutional neural network (CNN) has been developed. It contains two external and six internal layers, including two convolution layers followed by max-pooling, a flatten layer and two fully connected dense layers (Fig. 1).

The mean absolute error (MAE) was chosen as the loss function. MAE is a measure of errors between paired observations

expressing the same phenomenon. It is calculated as the sum of absolute errors divided by the sample size:

**Formula. 9.**
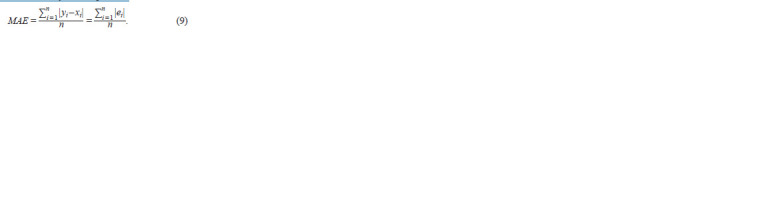
Formula9

The optimization strategy was based on the Adaptive Moment Estimation algorithm (ADAM). It combines both the idea of accumulation of movement and the idea of a weaker update of weights for typical signs. It is one of the most popular adaptive step-size methods (Kingma, Ba, 2014).

Gradient descent (CD) is a method that uses the fixed-point method to zero out the first derivative of the cost function, but it creates difficulties in complex applications

Estimation of the model’s precision. The accuracy of the model was estimated using the coefficient of determination (R2). The coefficient of determination is the proportion of variance of the dependent variable explained by the dependence model in question, that is, the explanatory variables:

**Formula. 10.**
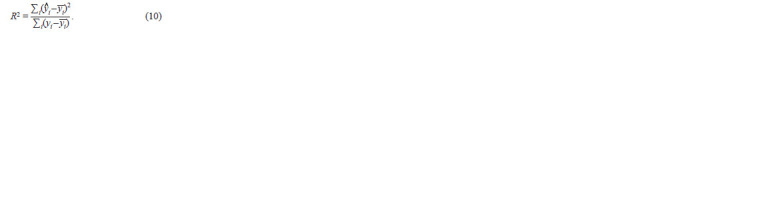
Formula10

Artificial noise in data. Small data size was modelled by first making a sample of a certain size with genotype quantities (integers) proportional to genotype probabilities calculated as described above and then normalized again to obtain frequencies. Thus, the smaller was the “sample size”, the bigger was the distortion. This procedure allowed us to obtain the training set of genetic setups similarly distorted.

Other sources of mistakes include diverse aberrations like misidentification of samples, size calibration errors in the course of fragment analysis, etc. It was modelled by making a vector of random values sampled from normal distribution with the average set to 0 and the standard deviation set to a desired value, and adding this vector to the vector of values delimiting different classes of ratios of individuals resulting from sexual or asexual reproduction.

## Results

A deep learning-based method for estimating the ratio of asexual and sexual reproduction in populations capable of switching between these reproductive modes has been developed. In its current form, the method is intended to use multiallelic traits, the most common of which are microsatellite repeats. The method achieved an accuracy value of 0.99. The method of training the neuron network appears to be critically important: our findings reveal that ignoring the variability in allele counts across genes and using uniform genotype matrices significantly reduces model precision. This underscores the importance of accounting for allelic diversity during training. In this regard, for each data set, the model was trained on a simulated data set of a similar dimension to a frequency matrix of the original data.

**Fig. 2.**
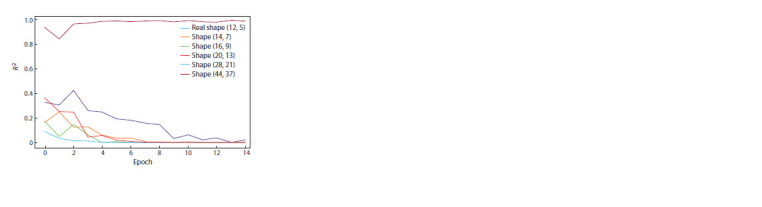
The dependence of the error value on the learning epoch for different dimensions of a frequency matrix of the training sample.

When the size of the training dataset matches the dimensionality of the actual data, the mean squared error converges to zero more rapidly compared to situations where the training dataset has a larger size (Fig. 2).

**Fig. 3.**
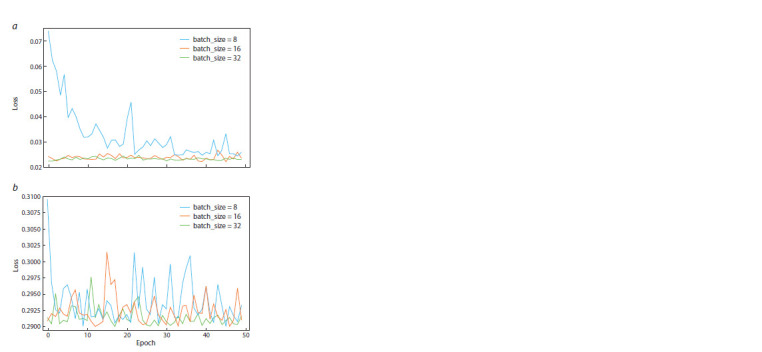
The dependence of the error value on the learning epoch for different sizes of the training sample and different learning rates. a – the graph shows the error value depending on the learning epoch, with a model learning rate equal to 0.01; b – the graph shows the error value depending on the epoch with a learning rate equal to 0.1.

With the model architecture chosen, the optimal number of learning epochs turned out to be 15, with the value of the number of epochs, the learning rate equal to 0.01 and the size of the training sample equal to 16, the minimal error value is achieved. With the learning rate of the model equal to 0.1, the error quickly takes a value less than 0.05 and does not rise above this value with the sample sizes of 16 and 32. With a learning rate of 0.1, the result is unstable, and the error value varies from 0.29 to 0.3 and does not drop below even with 50 training epochs (Fig. 3).

**Fig. 4.**
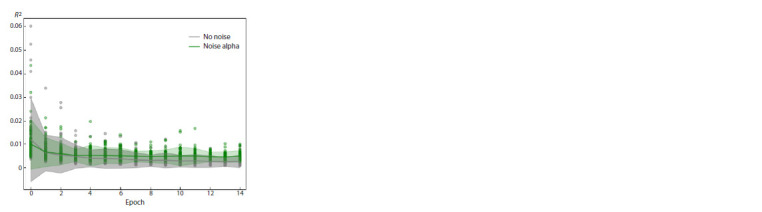
The effect of noise in the frequency of occurrence of a combination of alleles on the prediction error of the model. The green color shows the error distribution when training the model on simulated data with artificial noise in the frequencies of occurrence of a combination of alleles having a Gaussian distribution with a standard deviation of 0.05. The grey color shows the error distribution when training the model on simulated data without noise.

When noise occurs in the frequency values of the ratio of sexual and asexual reproduction, which may indicate errors occurring during sequencing, the average error values when noise occurs are higher than without noise, but with a standard deviation value of 0.05 differ by no more than 0.01 (Fig. 4). This computational experiment tests the method’s resistance to noise caused by sequencing errors.

**Fig. 5.**
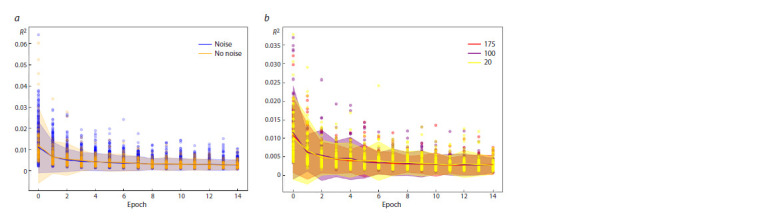
The dependence of the error value on the epoch of model training with a limited sample. a – the blue color shows the error distribution when training the model on simulated data containing noise in the form of limiting the sample to the training data and rounding the value of the sum of frequencies in a sample size from 10 to 200, followed by averaging the frequency of occurrence of a combination of alleles. The orange color shows the error distribution when training the model on simulated data without noise. The lines are depicting approximation curves for data with and without noise. The confidence intervals are shown in translucent color; b – the red color shows the error distribution for a sample size of 20 out of 200 possible; the purple color shows the error distribution for a sample size of 100 out of 200 possible; the yellow color shows the error distribution for a sample size of 175 out of 200 possible.

As the number of individuals in the sample increases, the confidence interval in the early epochs of model learning decreases. When comparing noisy data by sample size and non-noisy data, it can be concluded that the error does not differ much; at the initial stages of model training, the confidence interval is larger, but at the end of model training, both the average and the confidence interval differ slightly (Fig. 5). This computational experiment tests the method’s resistance to noise arising from limited sampling for analysis. The model was tested on experimental data, and values. The models obtained as a result of calculations coincided with the experimental data.

## Discussion

The model proposed here does not take into account a set of complications quite common in the real-world data. Different loci are often inherited dependently due to topological associations in chromosomes which per se may be of positive selective value and may change the expression level of some genes. These associations may be supported by assorted mechanisms bringing even distant loci physically together. Also, many microsatellites are organized in a more complex way than just a simple repeat of short sequences; in this case, the inheritance of microsatellite alleles may be distorted by non-allelic mutations in the adjacent areas of the genome. These accomplishments become a serious challenge when setting up models, which in turn may cause an unnaturally high level of mistakes in models or the necessity to develop models with many more parameters. This increases the numbers of model parameters’ computational time and complexity (Putman, Carbone, 2014).

The advantage of our convolutional neural network compared to traditional approaches is the ability to efficiently extract and process information from multidimensional data structures, which is critically important when analyzing genetic data. In particular, this means an increased ability to recognize complex relationships and mechanisms, which can provide more accurate predictions and a better understanding of genetic interactions. The model proposed is characterized by a high degree of accuracy, it is trained on data, the size of which exactly corresponds to the size of experimentally obtained genetic matrices, thereby minimizing the risk of overfitting, which often occurs when using larger, artifact datasets. This approach made it possible to achieve a significant level of accuracy already at the initial stages of training, which indicates the high efficiency of model training and its ability to quickly adapt to new data.

When training with data that are selected depending on the structure of the actual analyzed data, the model quickly reaches an accuracy of 0.95, after which the overtraining of the model does not happen. Overtraining of a neural network may not occur in some cases, for example, when a linear perceptron is used. In this case, all the minima of the error function are approximately equivalent to the desired point of the global minimum, thus overtraining cannot be achieved.

Noise in the data has a significant impact on neural network prediction results, especially in the cases of analyzing biological data such as DNA sequencing (Kircher, Kelso, 2010). Errors in data acquisition may be due to diverse reasons such as insufficient sample quality: poor quality of DNA or RNA, for example, due to degradation or contamination, can lead to errors in sequencing (Levin et al., 2020), and so can faults in sequencing technology (different sequencing methods have their own limitations and sources of errors). For example, some technologies may have difficulty with repeating sequences or with long DNA fragments (Adiconis et al., 2013). The very PCR process may become a source of noise: when samples are amplified using polymerase chain reaction (PCR), errors can occur, which are then transmitted to the sequencing results (Hsiao, 2019).

Frequency values of the ratio of sexual and asexual reproduction are subject to random deviations. This may make it difficult for the neural network to correctly identify patterns and dependencies. Incorrect or distorted data can cause the model to make incorrect assumptions about the distribution of data, which reduces its generalizing ability

However, as the results of the present experiment show, with a noise standard deviation of no more than 0.05, which roughly corresponds to the real situation, the difference in predictions is only 0.01. This indicates that the proposed method is sufficiently resistant to frequency noise that occurs during the acquisition of real data

Modern approaches, such as the use of model ensembles or techniques for estimating the uncertainty of predictions, can also help to effectively deal with noise (Zhou, 2025). This is especially true in biological research, where data may be distorted due to a large variety of reasons. The reasons are not specified in this work, since the noise level in the frequencies, when receiving real data, which are further analyzed, often does not exceed 0.05.

The noise caused by a limited sample size may result in an increase in the prediction error, and its negative impact can be mitigated by using a sufficient sample size. The observed decrease in the confidence interval with an increase in the number of objects in the sample indicates an increase in the accuracy of the model’s predictions as more data are accumulated. A comparison of error distributions in noisy and nonnoisy data at different stages of training shows that although the confidence interval for noisy data is wider at the initial stages, the error differences become less significant at later stages of training. This may indicate that with an increase in the number of training iterations, the model is able to adapt to noise and adjust its predictions. It is also worth noting that the simulation results agree with experimental data, which confirms the adequacy of the proposed method and its resistance to noise arising from a limited sample size. This opens up the possibility for applying this approach in various fields were working with noisy data is an everyday task, such as genetic research, medical diagnostics, and other scientific fields that otherwise would require the analysis of a larger amounts of complex data.

## Conclusion

Application of the described approach has its limits since violations of the equilibrium frequencies of genotypes can arise for a number of reasons not related to reproductive strategy, from genetic drift to sudden demographic changes. Therefore, in each specific case, it is necessary to involve external knowledge regarding the biology of the organisms under study. Further studies of populations with a mixed reproductive strategy and, accordingly, methods for detecting the characteristics of their genetic diversity should take into account, firstly, the inconstancy of the ratios of strategies in a number of generations, and secondly, possible sharp demographic fluctuations. The combinations of these two factors result in unusual patterns of genetic diversity.

## Conflict of interest

The authors declare no conflict of interest.
